# Efficacy of non-wearable VR-based behavioral training for preschool children with high-functioning autism spectrum disorder: a protocol for an upcoming randomized clinical trial

**DOI:** 10.3389/fpsyt.2025.1575695

**Published:** 2025-06-13

**Authors:** Naichi Kuo, Yukai Yao, Chenhuan Ma, Liting Chu, Jinjin Chen, Chunling Wan, Dandan Wang, Dingjie Lu, Xiaoqian Li, Yu Wang

**Affiliations:** ^1^ Department of Rehabilitation of Child Health Care Medical Division, Shanghai Children’s Hospital, School of Medicine, Shanghai Jiao Tong University, Shanghai, China; ^2^ Department of Child Health Care Medical Division, Shanghai Children’s Hospital, School of Medicine, Shanghai Jiao Tong University, Shanghai, China; ^3^ Institute of Child and Adolescent Health, Shanghai Municipal Center for Disease Control and Prevention, Shanghai, China; ^4^ Bio-X Institutes, Key Laboratory for the Genetics of Developmental and Neuropsychiatric Disorders, Ministry of Education, Shanghai Jiao Tong University, Shanghai, China

**Keywords:** autism spectrum disorder, virtual reality, early intervention, cognitive behavioral therapy, preschooler

## Abstract

**Objective:**

Autism spectrum disorder (ASD) is a neurodevelopmental condition with limited effective treatment options, including Applied Behavior Analysis, Transcranial Magnetic Stimulation, and certain medications aimed at managing associated behavioral and emotional regulation challenges. Primary interventions include educational training and behavioral modifications. However, long-term, intensive, and individualized rehabilitation training strategies are lacking, and new rehabilitation tools therefore urgently need to be developed. Virtual reality (VR) is a promising digital rehabilitation tool that may support the development of essential life skills, such as social communication and daily living abilities, in many children and adolescents with ASD. Herein, we introduce a protocol for an initial multicenter randomized controlled trial focused on preschool-aged children with ASD. This trial aims to investigate the clinical efficacy of behavioral training for young children with ASD utilizing VR technology.

**Methods:**

A total of 160 children aged 4–7 years with high-functioning ASD will be assigned to either the trial or control group in a 1:1 ratio. The trial group will undergo behavioral training based on VR technology and early family intervention guided by the rehabilitation team, whereas the control group will undergo early family intervention guided by the rehabilitation team. Both arms will undergo twice weekly sessions of either the trial or control intervention modality performed over 12 consecutive weeks. Outcome assessments will be performed at the start of the trial, throughout the intervention phase, and at follow-up intervals during the study.

**Discussion:**

The primary aim of this trial is to determine the clinical effectiveness of cognitive training using VR technology in children with ASD.

**Clinical trial registration:**

Chinese Clinical Trial Registry identifier, ChiCTR2400094189.

## 1 Introduction

Autism spectrum disorder (ASD) is a complicated neurodevelopmental condition characterized by several core symptoms, including social difficulties, restricted interests, and repetitive, stereotyped behaviors (1). The prevalence of ASD is on the rise, with roughly 1 in 36 8-year-old children in the United States currently meeting the diagnostic criteria for ASD (2). Conversely, the prevalence of ASD among children aged 6–12 years in China is around 0.7% (3). Children with ASD generally face a challenging prognosis, with a restricted ability to live, learn, and work independently as adults. Individuals diagnosed with ASD commonly encounter significant challenges in achieving independence in adulthood, impacting their capacity to live, learn, and work independently. Zhao et al. previously conducted research examining the economic impact of ASD in China. In this study, the overall annual cost of ASD in 2020 was calculated as $6,706.44 per person. Further, by calculating the prevalence of ASD among the population of China, it was estimated that approximately 6,233,127 individuals have ASD in the country. Based on these values, an estimated economic burden of $41.8 billion per year was calculated, indicating that ASD represents an important public health issue (4).

ASD is a lifelong condition with limited effective treatment options, for which the main interventions are currently based on education and behavior modification (5). However, most therapeutic institutions (e.g., speech therapy, psychotherapy, game therapy) require high-quality professionals and therapeutic scenarios, and they are often concentrated in large cities, where treatment is costly (6). Overall, treatment costs at city-based facilities are prohibitively high, posing a significant barrier to timely treatment access among children from low-income families (7).

Recent research has presented substantial evidence highlighting the wide therapeutic potential of digital health interventions (DHIs), including computer-assisted therapy, wearable technology, and smartphone applications (8). Compared to traditional intervention models that require offline one-on-one sessions with an expert, the use of DHI technology can scale up services at a relatively low cost, thereby overcoming many of the problems of traditional therapies, including high cost, difficulty to access, inefficiency, and lack motivation (9). Virtual reality (VR) technology, as a type of DHI, utilizes advanced computer technology to develop immersive virtual environments that integrate realistic visual, auditory, and haptic sensations where users can interact with objects in a virtual world in a natural manner using assistive devices. Further, they can interact with each other, thereby creating the feeling and experience of existing in the real environment (10). Previous studies have developed many VR systems for application to improve social skills (11), life skills (12), and social adaptability (13) in children with ASD. The findings from these studies have shown that children can understand, interact with, and respond appropriately to virtual environments, with the potential for these skills to generalize to real-life situations (14).

In comparison to traditional intervention models, VR technology has many advantages, including that it offers absolute control over input stimuli and environmental factors, provides a safe place for learning rules and repetitive training, and further reduces the need for real-world social interactions. These benefits can help alleviate sources of anxiety in children with ASD, thereby addressing their psychological needs (15). Children with ASD often show superior visual information input and processing skills, while their thinking can be characterized by visualization and concreteness (16). VR technology provides an optimal method of presenting information to attract the attention and interest of children with ASD. Further, VR learning scenarios can be designed to replicate various real-world situations, thereby allowing students to rehearse and implement their skills in a focused setting (17). This technique thus represents an effective method of presenting information to attract and maintain the interest of children with ASD. Finally, VR technology can facilitate the simultaneous training of multiple patients for interventions, while its integration with the internet can enable the performance of teletherapy, thus reducing the number of offline interventions and medical costs for families of children with ASD (18).

Early intervention training has been demonstrated to notably enhance developmental, behavioral, and core symptoms among children with ASD, resulting in positive physical, social, and emotional developmental outcomes, in addition to increased verbal and nonverbal communication abilities, which is crucial in influencing the prognosis of children with ASD (19). Nevertheless, the majority of previous intervention training protocols utilizing VR technology in children with ASD have concentrated on patients aged from 5-15.5 years. Overall, the majority of studies have concentrated on school-age and adolescent populations, with fewer studies investigating the impact of such interventions on preschool-age children (14).

The current state of VR technology presents several challenges. First, the majority of current VR systems employ head-mounted displays (HMDs), which rely on spectacle-like displays that obscure the user’s vision to present a three-dimensional view of the scene. However, this approach can result in adverse effects in some participants, such as cybersickness, a physical condition marked by symptoms like eyestrain, headache, dizziness, and nausea (20). In some cases, the use of these devices can also lead to anxiety and mood swings in children (21). Consequently, it is generally advised that head-mounted devices not be utilized by individuals under the age of 7, as the visual centres of these targeted individuals have not yet developed to the point where they are capable of effectively utilizing such devices (22). Additionally, younger children are more susceptible to training unsustainability, due to the size and weight of the devices, which can cause mobility problems and lead to physical discomfort. Finally, the inability of younger children to comprehend the precise operation and regulations of complex wearable VR technology can impede their ability to utilize these systems effectively, which can subsequently affect the efficacy of training (23).

To overcome these shortcomings of wearable technologies in the field of VR, we designed a behavioral training system based on VR technology to establish a digital intervention training platform. This system comprises three components: a nonwearable VR interactive game, a real-time detection system, and a cloud platform for assessment and management. This system was designed with the aim of enhancing cognitive functioning among children with ASD.

The diagnosis of ASD relies predominantly on behavioral observations and scale assessments, which are both time-consuming and inefficient. Furthermore, this process is susceptible to various external factors, including the psychological state of patients with ASD, cultural background of caregivers, experience of evaluators, and other variables. Consequently, these methods are prone to subjectivity and bias, which can result in a high rate of missed diagnoses. Therefore, the current protocol was designed to utilize both traditional psychometric scales and functional near-infrared spectroscopy (fNIRS) to evaluate changes in cerebral function in preschool-aged children with ASD prior to and following intervention. The fNIRS method employs near-infrared light (650–950 nm), which penetrates biological tissues to non-invasively monitor cortical activity. fNIRS is a non-invasive method for imaging the brain that monitors cerebral cortical activation by measuring the levels of two hemodynamic parameters: oxygenated haemoglobin (oxy-Hb) and deoxyhaemoglobin (de-Hb). These parameters allow the generation of a comprehensive picture of brain function (24).

This protocol describes an upcoming randomized, controlled, parallel-group, non-inferiority trial utilizing digital technology to implement a behavioral training system based on VR technology in preschool-aged children with ASD. The protocol includes subgroup randomization in conjunction with a 1:1 allocation, to ensure the random allocation of 160 participants to the two groups. The intervention group will receive cognitive behavioral training based on VR technology, in addition to early family intervention under the guidance of the rehabilitation team. Conversely, the control group will receive only early family intervention under the guidance of the rehabilitation team. The effectiveness of cognitive-behavioral training utilizing VR technology in children diagnosed with ASD will be explored by analysing the effects of different modes of rehabilitation training. Additionally, resting-state fNIRS will be used to objectively assess pre- and post-intervention functional brain changes.

We hypothesized that the intervention group would exhibit a potentially positive impact on ASD-related core symptoms, in terms of scale scores and brain function data compared to the control group.

## 2 Methods and analysis

### 2.1 Study settings

**Figure 1:**
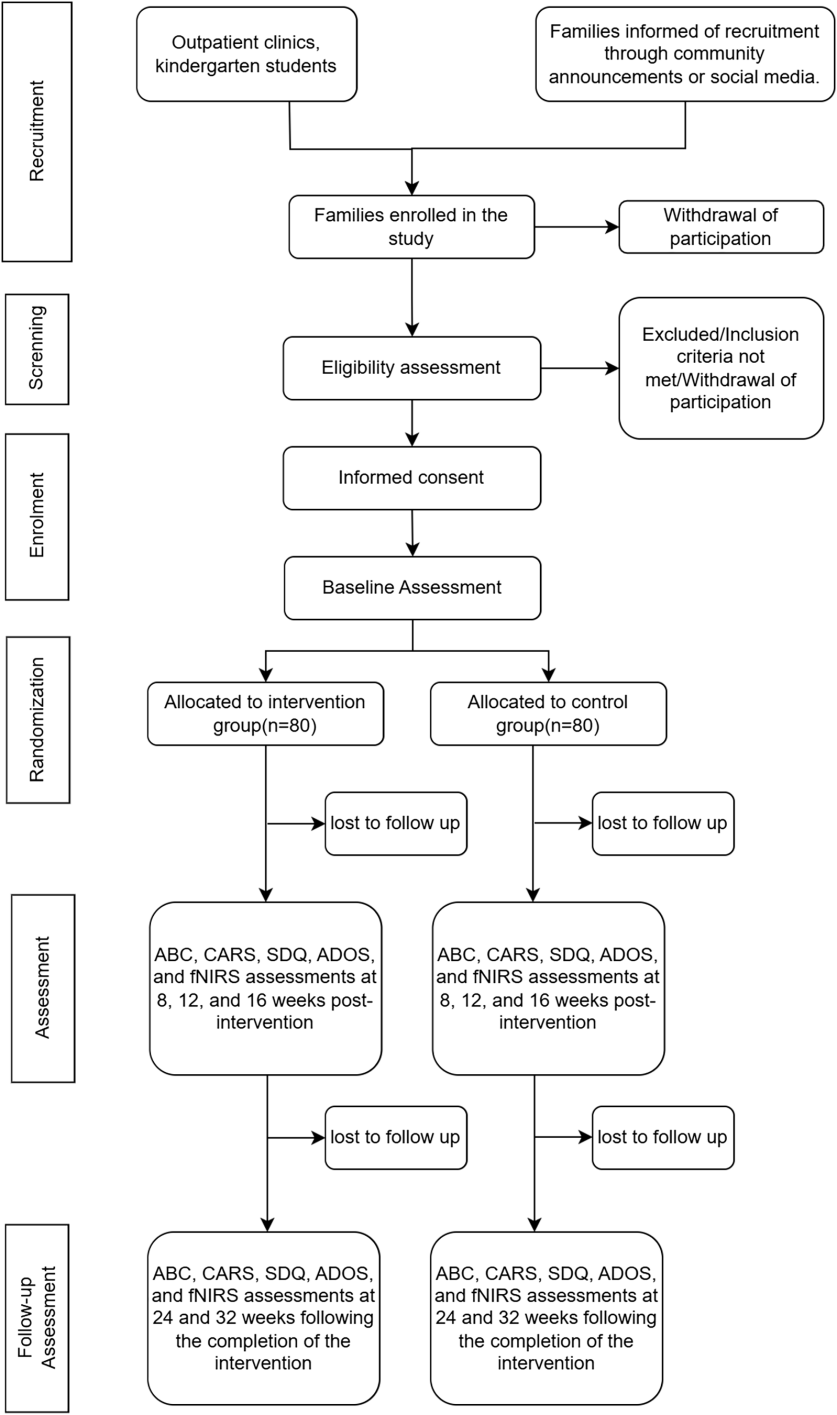
Protocol flow chart of the study. ABC, Autism Behavior Checklist; CARS, Child Autism Rating Scale; SDQ, Strengths and Difficulties Questionnaire; ADOS, Autism Diagnostic Observation Schedule; fNIRS, functional Near-Infrared Spectroscopy.

This study aims to offer a thorough overview of digital rehabilitation research methodologies for ASD interventions, which will be achieved by comparing the intervention effects of a trial group (cognitive-behavioral training based on VR technology and early home intervention guided by a rehabilitation team) to those of a control group (early home intervention guided by a rehabilitation team). This study is planned as a randomized, controlled, parallel-group, non-inferiority trial with an equal allocation ratio between intervention and control groups. An overview of the protocol is illustrated in **Figure 1**.

Participants were enlisted through advertisements in outpatient clinics, kindergartens, community bulletins, and social media platforms. Overall, 160 children aged 4–7 years who met the criteria for ASD, according to the criteria outlined in the Diagnostic and Statistical Manual of Mental Disorders, Fifth Edition (DSM-5), were enrolled. The formal diagnosis was determined using the gold standard instrument, the Autism Diagnostic Observation Schedule, Second Edition (ADOS-2), alongside clinical judgment by experienced psychologists. All participants were between the ages of 4 and 7 years old. Only children who satisfied the eligibility criteria (IQ score of at least 70) for the study were enrolled after completing the Wechsler Preschool and Primary Intelligence Scale (Chinese version) assessment, administered by a professional clinical psychologist. The randomization process was performed using SAS software (version 9.4, SAS Institute).

The primary objective of this study was to create and apply a behavioral training system using VR technology for preschool children with ASD. This system comprises a program utilizing VR technology, a real-time computer monitoring system, and a cloud-based management system. Participants in either the experimental or control group will receive 24 sessions of the intervention model (two sessions per week for 12 consecutive weeks). Participants will be assessed at six time points as follows: before the start of treatment; at 8, 12, and 16 weeks during the treatment period; and at 24 and 32 weeks following the completion of the intervention. The evaluation will follow the guidelines of good clinical practice. The assessment will include questionnaire scales completed by parents, observational scales administered by clinicians, and quantitative fNIRS to measure changes in hemoglobin (Hb) concentration at rest. These include indicators of training, behavioral symptoms, symptom severity, prosocial behaviors, and brain functioning.

The overall aim of this assessment is to compare and evaluate outcome differences between the two groups based on the intervention model results. Thus, we plan to assess the effectiveness of the behavioral training system using VR technology for preschool children with ASD. Concurrently, the intervention process will employ real-time data based on Continuous Performance Testing (CPT) technology to monitor changes in the training effects on the children.

### 2.2 Sample size calculation

The sample size was determined based on the primary outcome measure and the scores from the ADOS scale. Previous studies reported an average ADOS-2 score of 16.9 ± 4.1 for children with ASD (25). In this study, we hypothesized a clinically meaningful difference of 2 points in the intervention group compared to the control group, indicating the efficacy of the VR-based intervention. The required sample size was calculated using the PASS 15.0 software, with the stipulation of α=0.05 and the test efficacy 1-β=0.8. This process yielded a sample size of 72 patients. Given a 10% loss of visits, the calculated sample size for each group was 80, with a total sample size of 160 cases.

### 2.3 Eligibility criteria

Inclusion criteria:

1. Children diagnosed with ASD who met the DSM-5 diagnostic criteria and were further confirmed using the gold standard tool, the ADOS-2, with a total score >7 required for study inclusion;
2. Age 4–7 years old;
3. IQ>70 based on Wechsler Preschool and Elementary Intelligence Scale (Chinese version);
4. Able to comprehend instructions with assistance from the researcher;
5. No recent history of participating in other training programs.

Exclusion criteria:

1. Children with features of ASD but with neurometabolic epilepsy or epilepsy as the aetiology;
2. Children with cerebral developmental disorders on cranial CT;
3. Children with hearing abnormalities on audiometry;
4. Children with Fragile X syndrome or other syndromes resulting from known genetic defects or inherited metabolic disorders;
5. Children with other neurological or psychiatric diseases, including intellectual disability without ASD, schizophrenia, language disorders, and social communication disorder.

### 2.4 Trial status

Currently, the trial status is recruiting. Inclusion of participants initiated on 24 December 2024. The anticipated end date is 30 September 2026.

### 2.5 Randomization and blinding

Participants will be assigned randomly to either the experimental or control group in a 1:1 ratio, with randomization lists constructed by an independent statistician using SAS version 9.4 software. Group assignments were concealed using sequentially numbered, opaque, sealed envelopes prepared in advance. Upon enrollment, research staff opened the next envelope in the sequence to reveal the participant’s group assignment. Due to the nature of the intervention, blinding participants or therapists to group allocation was not feasible. However, to reduce assessment bias, all outcome evaluations will be conducted by independent assessors who are blinded to group assignments. To further ensure the reliability of outcome measurements, inter-rater reliability will be evaluated using intraclass correlation coefficients for clinician-administered observational tools such as the ADOS-2 and CARS. The results of the trial, including the patient’s identifying information and details of the intervention, will be de-identified. Final analyses will be performed by a statistician who remains blinded to group allocation.

### 2.6 Ethics approval and informed consent

This randomized controlled trial was carried out under the guidance of Shanghai Children’s Hospital in China. Parents or guardians will be required to provide written informed consent before participating in the study. Participants will further be allowed sufficient opportunity and time to inquire and consult with their friends and family before making a final decision. Furthermore, parents and caregivers will ensure that their children’s treatment is not adversely affected, regardless of whether they participate in the experiment or not. It is also important to emphasize that participation in the experiment is entirely voluntary and that individuals maintain the right to drop out at any time. Further, all experimental steps will be preceded by obtaining written informed consent. The principal investigator or co-investigators must have an impartial witness present during the signing of informed consent forms. Due to the young age of the subjects (ranging from four to seven years old), it was deemed unnecessary to obtain personal consent from the patients themselves.

### 2.7 Evaluation process

To ensure that participants meet the study criteria, the Wechsler Preschool and Primary Scale of Intelligence (Chinese revision) test was conducted before the study began to comprehensively assess the children’s overall intellectual ability. To evaluate how effective behavioral training using VR technology is in the rehabilitation of children with ASD, the following instruments will be administered: the parent-rated autism behavior checklist (ABC) to assess behavioral symptoms, Child Autism Rating Scale (CARS) to evaluate the severity of symptoms, Strengths and Difficulties Questionnaire (SDQ) to assess prosocial behavior, and Autism Diagnostic Observation Scale-2 (ADOS-2) to assess the child’s interactional behaviors. Brain function will further be assessed via the application of the fNIRS technique, which employs near-infrared light to gauge changes in cerebral blood flow. To ensure the authenticity and reliability of the data, parents received detailed instructions on how to complete the questionnaires, and all responses were reviewed for completeness at the time of collection. Any incomplete or questionable responses were verified and corrected in consultation with the parents. To enhance consistency and reduce variability, the same clinician and parent will complete the pre- and post-intervention assessments for each participant.

#### 2.7.1 Wechsler Preschool and Primary Scale of Intelligence (Chinese version)

The WPPSI is a psychological tool used to assess the cognitive abilities of children aged 36 to 95 months. The WPPSI will primarily be used to establish a starting point for the children involved in the study (26). This test comprises 15 subtests which assess children’s overall cognitive functioning, including general knowledge, missing places, and vocabulary. This assessment includes the Verbal Comprehension Index (VCI), Visuospatial Index (VSI), Fluid Reasoning Index (FRI), Working Memory Index (WMI), and Processing Speed Index (PSI), which collectively assess intelligence (27).

#### 2.7.2 Parent-rated Autism Behaviors Checklist

This scale assesses the behavioral status of children and comprises 57 items pertaining to the characteristics of children with ASD. These include five aspects: sensation, interaction, body and object use, language, and social life self-care. Each item receives a score ranging from 0 to 4 on a 5-point scale, yielding a total possible score of 158 points. A score of ≥62 points is sufficient for the diagnosis of ASD, with higher scores suggesting more severe associated behavioral symptoms (28).

#### 2.7.3 Child Autism Rating Scale

This scale has previously been employed to evaluate symptoms of ASD in children diagnosed with ASD aged 2 years or older. This 15-item scale is designed to assess behaviors across 14 domains that are commonly impacted by severe issues in ASD, as well as an overall impression. Each item is scored on a 4-point scale. A total score below 30 indicates that the participant does not have ASD. Scores between 30 and 36 indicate mild to moderate ASD, while scores of 36 or higher indicate severe ASD (29).

#### 2.7.4 Strengths and Difficulties Questionnaire

This scale is predominantly used to evaluate emotional and behavioral problems in children and adolescents. The scale comprises five subscales: emotional symptoms, conduct problems, hyperactivity and attention deficit, peer relationships, and prosocial behavior. The first four subscales comprise the Difficulties Questionnaire, which assesses negative emotions and behavioral problems. In contrast, the Pro-Social Behavior subscale serves as the Strengths Questionnaire, which reflects positive behaviors. A higher score on the Difficulties Questionnaire indicates a more severe problem, whereas a higher score on the Strengths Questionnaire indicates more positive behavior. This scale has previously been utilized to evaluate prosocial behavior in children with ASD (30).

#### 2.7.5 Autism Diagnostic Observation Schedule, Second Edition

ADOS-2 is a semi-structured assessment tool designed to evaluate the interactive behavior of children. This scale comprises four modules, with module 2 focusing on children who have limited language abilities and are unable to communicate in an efficient and fluid manner, primarily within the context of preschool education. Each module comprises five items and assesses communication, social interaction, playing with objects using them imaginatively, as well as individual stereotypical and repetitive behaviors (31). Each item is rated on a scale from 0 to 2, where 0 represents typical performance and 2 indicates abnormality. Higher scores indicate more severe symptoms (32).

#### 2.7.6 Resting-state fNIRS

A near-infrared quantitative brain function imaging device (model: The NirScan-6000A, manufactured by The Danyang Huichuang Medical Equipment Co., Ltd.) will be used to measure changes in hemoglobin (Hb) concentration at rest. This device non-invasively monitors oxygenated hemoglobin (HbO2), deoxygenated hemoglobin (HbR), and total hemoglobin (HbT) by analysing changes in light intensity. It is equipped with 24 source transmitters and 24 receivers, allowing for up to 48 measurement channels. These channels cover six brain regions on each side: the frontal, parietal, and occipital lobes. The left premotor cortex, right premotor cortex, left prefrontal cortex, right prefrontal cortex, left parietal lobe, right parietal lobe, left occipital lobe, and right occipital lobe were selected as the regions of interest (ROIs). Near-infrared light at three wavelengths (730, 808, and 850 nm) is emitted by each source, and detected by nearby receivers after passing through the brain’s cortex. Using the Beer-Lambert Law, the device calculates Hb concentration changes by assessing near-infrared light absorption in each channel (33).

All fNIRS brain function tests will be conducted in a quiet environment free from noise and other distractions in a designated testing room. During the resting phase, the child will be required to sit upright, maintain a state of mental relaxation, and keep their head as motionless as possible. During measurement, participants will wear a cap on their heads, adjust the probe, and conduct the test, which involves switching on all channels to measure changes in blood and oxygen signals of the cerebral cortex in the resting state as well as recording the spontaneous brain activity of the subject.

### 2.8 Interventions

#### 2.8.1 Behavioral training based on VR technology

The intervention group will participate in 12 weeks of behavioral training targeted to preschool-aged children with ASD based on VR technology developed by the researchers. This training will be conducted twice a week for one hour. The same therapist will accompany each child during each intervention session. A comprehensive course schedule is provided in **Supplementary Table 1**.

**Figure 2:**
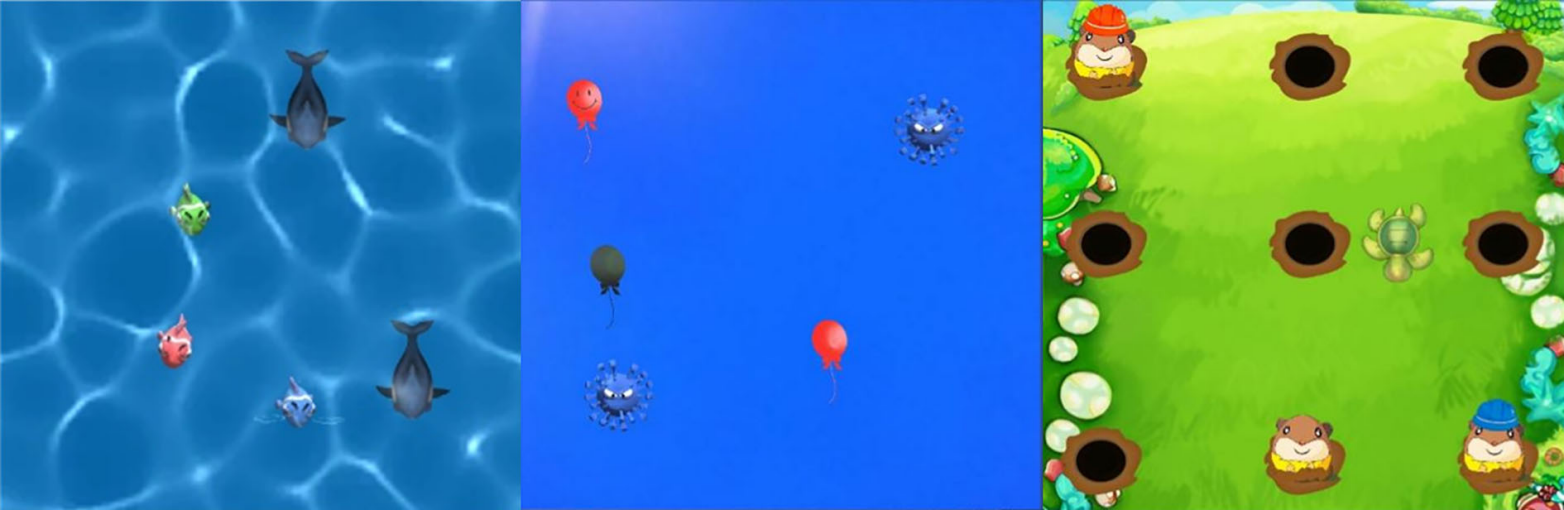
Overview of the three games in the Behavioral Training System for preschool children with ASD based on VR technology.

The intervention will use nonwearable VR technology, including a desktop computer (Lenovo) with an integrated LED interactive floor screen (3m*3m), and a resolution of P3.91. The nonwearable technology comprises a computer display projected onto the tile screen on the floor. Background music and game prompts are played simultaneously via an audio system, creating an immersive and interactive gaming experience. Cognitive-behavioral training based on VR technology comprised 4 steps (**Supplementary Table 2**) and 3 game scenarios (**Figure 2**). The training requires children to complete multiple tasks simultaneously, and utilizes an adaptive system that enables children to navigate between different scenarios. This process was designed to alleviate cognitive deficits in children. In scenes that combine both visual and aural stimuli, such as underwater worlds, country gophers, and aerial balloons, the perception of specific targets and the subsequent performance of post-perception motion tasks are performed simultaneously. In such scenarios, the child is required to respond to a specific target while ignoring distracting stimuli as they navigate an interactive ground screen to alter their position and engage with the intended target, while avoiding the unintended target. The procedures and images of the games in this intervention are presented in **Supplementary Figures 1** and **2**. The game’s difficulty is modified in real-time according to the child’s performance, thereby ensuring continuous and appropriate challenges at a preset difficulty level that is engaging yet manageable.

For each participant, a unique patient ID is generated, and the number of interventions will be recorded. Concurrently, the interactive ground screen collects real-time data on the children’s interactions, records scores, and analyses the children’s training indicators based on real-time CPT data. At the conclusion of each training session, the data will be transferred to a cloud-based data management platform, where it will be stored for later analysis. This will allow trainers to systematically and objectively customize training content.

#### 2.8.2 Early family intervention

Early family intervention will adhere to the principles of child development. This intervention will entail a comprehensive assessment of each child and the formulation of an intervention program accordingly. The assessment will commence with an examination of each child’s neuropsychological development with the developmental characteristics of ASD. Subsequently, an assessment of the developmental level of children with ASD will be carried out across various domains. The assessment will further encompass an analysis of the family environment and all available resources. Based on the present analysis, an individualized education plan prioritizing social interaction as the core training content and utilizing games as the basic carrier will be formulated. Early family intervention will prioritize the family as the centre of intervention; as such, parents will be the primary implementers of the treatment, with therapists acting as supervisors. The therapist assumes the role of supervisor for the parents and oversees the implementation of the IEP at home. Parents will receive one hour of intervention training per week organized by the therapist. In addition, one hour of training per week will be conducted at home, in conjunction with the classroom video provided by the therapist. The intervention duration will be 12 weeks for each group. Weekly telephone and monthly outpatient follow-ups will be conducted to monitor the implementation of the parental intervention.

### 2.9 Settings

The VR-technology-based intervention in the trial group will be implemented offline at each hospital, whereas the control group will undergo early home intervention after the parents are trained. Both groups will be required to undergo early home interventions guided by a rehabilitation team. A gradual approach to early family interventions will be provided by therapists (licensed psychologists and speech therapists) trained in performing early family interventions. These therapists will introduce concepts and strategies, demonstrate their use, and guide the parents in practicing them. The instructions provided by the therapist to each parent-child dyad will remain consistent throughout the study.

### 2.10 Data management

The information obtained for this project will not be made available to any individual outside of the research team, with the exception of the investigators, to maintain confidentiality and personal safety. During the clinical trial and data processing phases, proper data management practices will be followed. All records, including original medical documents, protocol copies, patient identifiers, source data, informed consent forms, case report forms, and other relevant documents, will be accessible. Medical records and related forms must remain unaltered without proper authorization. Any necessary corrections should be made as additional statements with justification, and must be signed and dated by the physicians involved. The destruction of research documents from the clinical trials is not allowed.

### 2.11 Risks and adverse events

Adverse events will be documented throughout the study, and an adverse event reporting system will be established following the guidelines set by the theoretical committee. In the case of serious adverse events, including death, life-threatening symptoms leading to hospitalization or prolonged hospitalization, congenital anomalies/deformities, and permanent or major disability, the investigator will be responsible for reporting the findings to the relevant ethics committee.

This study will concentrate on applying non-wearable VR technology and home-based interventions to enhance core symptoms in children with ASD. The planned intervention does not involve any invasive procedures, and will be delivered through an interactive floor screen or home intervention model, which are unlikely to cause any physical side effects. Additionally, the assessments planned to be used in this study are widely utilized in clinical settings, and are regarded as relatively low-risk owing to their non-invasive characteristics. Previous studies have indicated that cybersickness is a prevalent adverse reaction to VR experiences, manifesting as fatigue, discomfort, and dizziness (34). Prior to beginning the intervention, participants’ susceptibility to cybersickness will be assessed using the Motion Sickness Susceptibility Questionnaire Short Form. Results from this questionnaire will guide an individualized risk assessment to identify children who may have heightened sensitivity to VR-induced discomfort. For those participants without prior experience in VR, introductory exposure sessions will be provided to facilitate gradual adaptation at an individualized, comfortable pace. During the intervention sessions, a trained therapist will closely monitor each participant’s response, promptly adjusting, pausing, or discontinuing sessions if symptoms of discomfort or distress occur. Persistent adverse responses will lead to the child’s withdrawal from the intervention to prioritize their safety and well-being. Additionally, session duration and VR exposure time will be strictly controlled during the intervention and research assessments to prevent potential negative effects stemming from excessive or inappropriate VR usage.

## 3 Statistical analysis

The fNIRS data will be analyzed using the NirSpark software package developed by HuiChuang in China. During preprocessing, the first 20 s of each recording will be removed to eliminate signal instability and ensure data quality. Motion artifacts will be identified and corrected on a per-channel basis using spline interpolation, and a band-pass filter (0.01–0.20 Hz) will be applied to reduce interference from general physiological noise. Optical density data will be converted to oxygenated hemoglobin (HbO2) concentration values based on the modified Beer–Lambert law, using age-adjusted differential path length factors of 5.5, 5.2, and 4.6 as appropriate (35). After preprocessing, a resting-state functional connectivity analysis will be conducted to examine connectivity between brain regions. The resting-state data for each subject and time point will be extracted, and the oxygenated hemoglobin levels in each brain region’s time series will be quantified. Pearson correlation coefficients will be calculated for oxygenated hemoglobin across channels and brain regions to assess connectivity. These coefficients, represented as r values, will be used as indices of whole-brain resting-state functional connectivity. To normalize the distribution, Fisher’s z-transformation will be applied to the correlation coefficients (r), producing transformed Z-values that represent the strength of connectivity for each channel. This should allow for comparisons of relative connectivity strength ROIs. Ultimately, focusing on the eight ROIs of the cerebral cortex, their resting-state functional connectivity intensities were analyzed in depth to reveal the functional interactions between different brain regions.

Data analyses will be performed using SPSS software (version 26; IBM Corp.). Paired-sample t-tests will be employed to assess for pre- and post-treatment comparisons between normally distributed measures. Similarly, the Kappa consistency test will be used to perform pre- and post-comparisons of count data. Independent sample t-tests will be employed for between-group comparisons of normally distributed measures, while nonparametric tests will be utilized for between- and within-group comparisons of non-normally distributed measures. Repeated ANOVAs will be conducted to evaluate the treatment effects within each group. The goal of this study is to identify any differences in variables between baseline and post-test measurements in both the treatment and control groups. The factors considered include time (before and after) and group (experimental vs. control), with each measure serving as a dependent variable. For measures that meet the normality test, means ± standard deviation will be used to describe results. Conversely, medians and percentiles will be employed for measures that do not meet the normality test. Count data will be presented as the median and interquartile range. Statistical significance will be defined as a p-value of 0.05 or less. To manage missing outcome data, we will assume that data are missing at random and will apply multiple imputation as the primary approach. Bonferroni correction adjustments will be applied where appropriate to control for the risk of Type I error due to multiple comparisons.

## 4 Discussion

Prior research has demonstrated that VR technology can enhance cognitive abilities, social skills, and life competencies in children with ASD (36). In contrast to the widely used wearable VR devices, this study adopts non-wearable VR technology, specifically selected to reduce physical discomfort and mitigate sensory overload. The VR scenarios included in our intervention are thoughtfully designed to be adaptive, interactive, and engaging, featuring dynamic difficulty adjustments to sustain children’s motivation and interest. To further support engagement and reduce dropout rates, therapists will provide individualized guidance and maintain consistent, ongoing communication with parents throughout the intervention.

As many children with ASD have sensory perceptual deficits, traditional intervention strategies conducted in different environments may heighten their negative emotions (37). Moreover, a significant number of children with ASD experience sensory integration disorders, which can hinder the effective processing of external sensory information in the brain, leading to uncoordinated body functions (38). In this study, non-wearable VR technology will be employed to eliminate specific external stimuli and simplify complex stimuli, thereby enabling children with ASD to focus on a specific scene and respond to specific stimuli. This technology offers a promising avenue for intervention training among children with ASD.

Cognitive-behavioral training with non-wearable VR technology aims to enhance the core symptoms of ASD and improve cognitive abilities, including sensory processing, motor skills, and response inhibition. In one initial clinical trial, VR technology was employed as an adjunctive therapeutic tool in conjunction with Learning Style Profile (LSP). Parental questionnaires, such as the ABC and CARS, were used to assess the efficacy of this approach. The results demonstrated that the VR-augmented treatment yielded more pronounced improvements in core symptoms associated with social deficits, repetitive stereotyped behaviors, and response inhibition in comparison to the control group (39).

In this study, we employed VR training as the primary intervention for the intervention group, and further introduced a two-player game mode to enhance social interaction opportunities. Additionally, the incorporation of pre- and post-intervention assessments is required to facilitate further to explore the effectiveness of interventions for children with ASD, as well as to extend the follow-up period to ascertain the sustainability of the observed effects. In the parent questionnaire, the SDQ will be used to assess the degree to which emotional problems have improved. However, this scale is inherently subjective when completed by parents alone. As such, the ADOS will be introduced by trained researchers to assess symptom improvement. This approach will help to standardize and codify behavioral observations using highly structured observations and tests, thereby enhancing the ability to detect core ASD deficits more effectively. Finally, fNIRS will be employed to assess brain function before and after the intervention, in order to evaluate the impact of VR-based training on brain plasticity, and to understand the neural mechanisms underlying behavioral improvement. To assess the sustainability of treatment effects, participants will complete follow-up evaluations at 24 and 32 weeks post-intervention. These extended assessments will help determine whether improvements in cognitive abilities, social communication, and daily living skills are maintained over time.

### 4.1 Limitations

This protocol primarily used three distinct cartoon-based scenarios of interactive play to improve core symptoms in children with ASD. The intervention content focused on general cognitive functions, including attention, response inhibition, and motor coordination, without including more complex or individualized skill domains. Additionally, the training did not target specific skills, and the limited variety of scenarios may reduce engagement over time owing to boredom or loss of novelty. These limitations highlight the need for more flexible and developmentally appropriate training modules that can be personalized to individual therapeutic needs. Future versions of the training system will incorporate a broader range of virtual environments simulating real-world social and adaptive tasks, such as peer interaction, independent eating, toileting, and emotional recognition, with the aim of enhancing communication and daily living skills.

Additionally, the human–computer interaction design of the system remains relatively basic, relying on pre-programmed stimuli and structured response formats. The current absence of real-time adaptability, multimodal feedback (e.g., voice, gesture, facial expression recognition), and context-sensitive interaction limits the naturalistic quality of the VR experience. Enhancing bidirectional interaction and responsiveness is essential for simulating authentic social exchanges and improving user engagement.

The application environment of VR interventions is another important consideration. This training is typically restricted to structured institutional settings, such as hospitals, which may limit accessibility and hinder the generalization of acquired skills to everyday life. To address this issue, future research should investigate the feasibility of portable, home-based delivery formats, such as projector-based systems or augmented reality technologies, enabling children to engage with therapeutic content directly within their natural environments. Integrating intervention tasks into familiar daily contexts may enhance ecological validity and facilitate real-time learning and behavioral transfer. Furthermore, increasing the adaptability of VR systems for in-home use could notably improve user adherence, caregiver involvement, and the long-term sustainability of intervention outcomes for children with ASD.

This study also only included children with an IQ of above 70, which may limit the generalizability of the findings to the broader ASD population, particularly individuals with more severe cognitive impairments. Future studies are warranted to explore the adaptability and effectiveness of VR-based interventions for children with lower cognitive functioning. Further research should also aim to address the limitations identified in this study.

### 4.2 Conclusions

In summary, we outline the protocol for a pioneering study aimed at exploring the feasibility and safety of non-wearable VR as a primary therapeutic approach for children with ASD. This cognitive-behavioral training system based on VR technology represents a novel digital therapeutic approach that is convenient, cost-effective, efficient, reproducible, and highly compliant. The system reduces the professional-level requirements for rehabilitation therapists to a certain extent, facilitates their popularization and application, and helps resolve the rehabilitation dilemma faced by many families raising children with ASD.
