# Supplementary material for: Efficacy of non-wearable VR-based behavioral training for preschool children with high-functioning autism spectrum disorder: a protocol for an upcoming randomized clinical trial
Source: Front Psychiatry. 2025 Jun 13;16:1575695. doi: 10.3389/fpsyt.2025.1575695 (PMC12202513; doi:10.3389/fpsyt.2025.1575695)
Supplement: Supplementary file 1 [file Supplementaryfile1.docx]

Supplementary Material

# Supplementary Figures and Tables

## Supplementary Figures

**Supplementary Figure 1. Images of cognitive behavioral training based on the nonwearable VR training program**


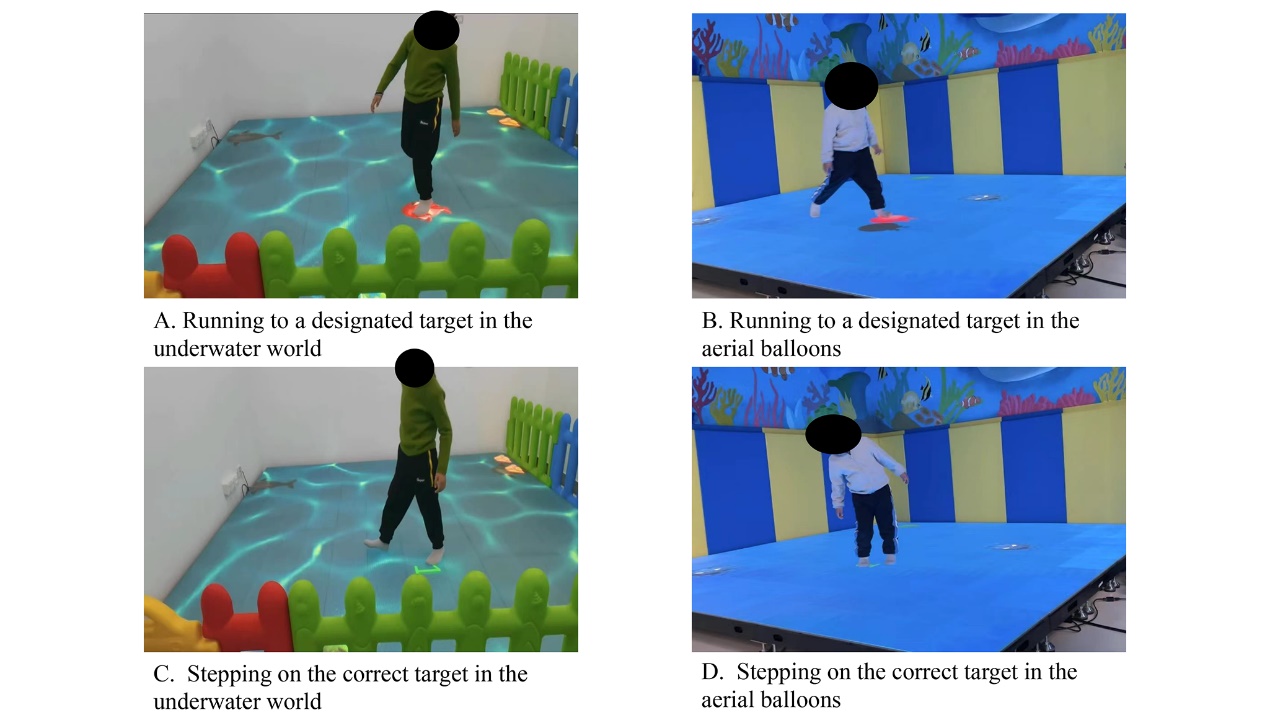


**Supplementary Figure 2. Images of cognitive behavioral training based on the nonwearable VR training program**


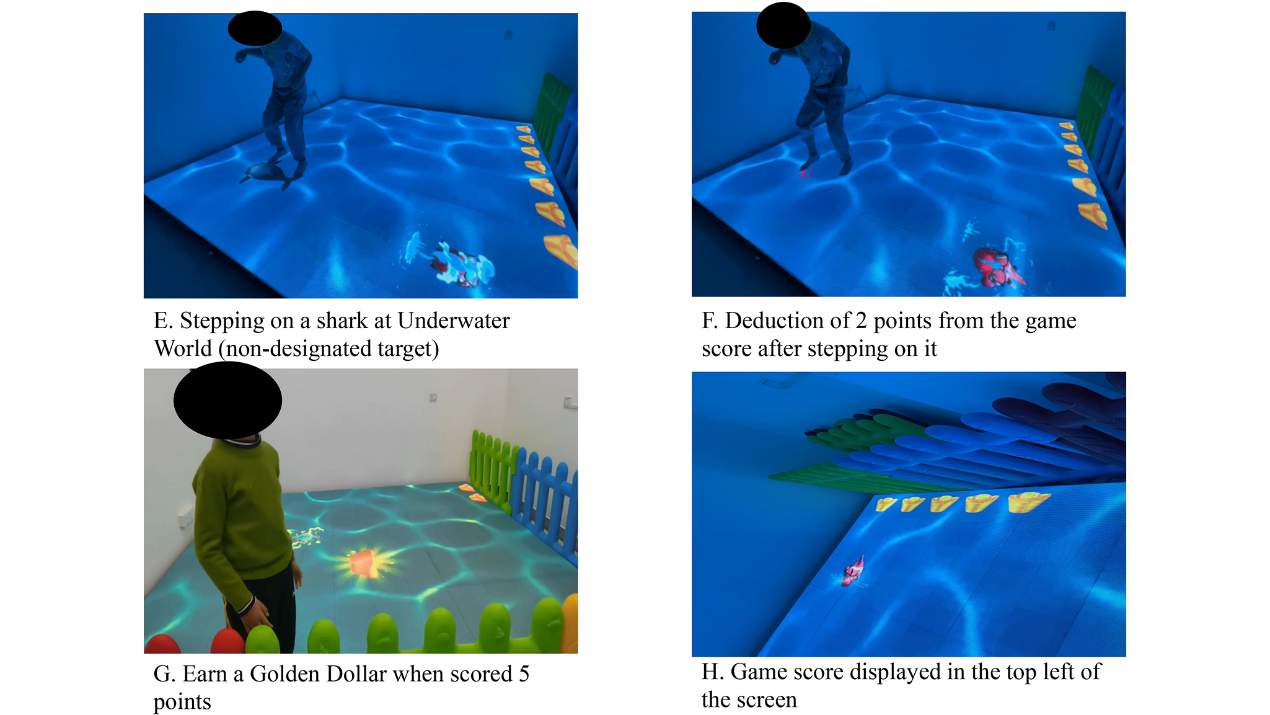


## Supplementary Tables

**Supplementary Table 1. Overview of cognitive-behavioral training based on the nonwearable virtual reality (VR) program curriculum.**

| **Week** | **Session** | **Goal** | **Content** |
| --- | --- | --- | --- |
| 1 | Pre-Intervention Assessment and Introduction to the Program | Familiarize participants with the program | 1. Provision of an overview of the program, including an explanation of the process, the basic rules, and the general use of the VR game. 2. Conduction of pre-intervention scales and brain function assessments to establish the pre-intervention baseline. |
| 2 | Observe and analyze the content and context of the game. | Guide participants through an immersive experience of the game | 1. The therapist guides the participant into the interactive screen area. 2. Children are encouraged to participate in the game, which helps them learn to pay full attention to external stimuli. |
| 3 | Rule Communication and Awareness | Ensure that all participants are fully aware of the game rules. | 1. The therapist conveys the rules of the exercise to the participants via the provision of concise verbal instructions and behavioural demonstrations. 2. Children are instructed to identify the target through visual observation and movement of their left and right feet following the receipt of initial instructions. |
| 4 | Gain familiarity and interact with one another | Guide participants to step on the correct target and score points | 1. When the participant approaches the correct goal, the virtual context provides positive reinforcement in the form of audio cues and the accumulation of bonus points, while the therapist simultaneously provides the participant with feedback. This ensures that the participant is able to recognize their performance through the therapist's facial expressions and verbal feedback. 2. The therapist also monitors the emotional changes of the participant in the game, creating opportunities for social interaction while participants progressively achieve their goals. |
| 5 | Learning to cope with negative behavioural experiences and negative emotions | Guide participants in learning to accept the outcomes of various in-game situations, such as rule infractions and failures in the game, and in recognizing the appropriateness of emotional responses | 1. In the event that a participant steps on the incorrect target, a beep and a red demerit is issued by the virtual situation, thereby indicating the error. If the participant fails to detect this error in the game, no points will be scored, and the therapist will be made aware of this fact during the process. 2. If the participant experiences negative emotions during the game, they will be instructed in the skills required to accept and cope with these emotions, and will be encouraged to make the appropriate decisions in light of these emotions. |
| 6 | Training in the spirit of play | Lead participants to communicate with each other and learn to wait and take turns | 1.Two participants will take turns working individually performing intervention training, during which participants learn to wait and take turns. 2.The therapist will repeatedly convey the importance of the turn-waiting rule when changing participants to foster good playfulness. |
| 7 | Social skills training | Collectively instruct participants on the completion of the game | 1. Pairs engage in a collaborative endeavour, in which participants disseminate rules and collaborate to fulfil the game objectives.  2. The therapeutic intervention will enhance the opportunity for verbal interaction and eye contact between participants throughout the game, aiming to enhance their socialization skills. |
| 8 | Observing and responding to conflict | Observe situations and conflicts in the game to learn social understanding | 1. The therapist will provide instructions on how to recognize and respond to conflict when it occurs in the two-person game. 2. The therapist will repeatedly remind participants of the correct rules of the game. |
| 9-11 | Conducting Air Balloon, Under the Sea, and Rural Gopher Games | Combination with the norms acquired in the pre-program (including targeted learning air balloons, underwater world, rural gopher) to strengthen the children's game performance | 1. In the game, pairs of players will take turns to play. 2. The two players play the game together. 3. Following repeated training of the same game, the therapist modifies the game scenario to stimulate the participants' attention and cognitive sensitivity. The participants maintain their motivation for training while applying the learned methods in a coherent manner in order to improve repetitive stereotypes while simultaneously enhancing the overall participation and freshness of the training. |
| 12 | Individualized training | Performance of intensive targeted training based on the performance of different participants' pre-existing backend data | 1. The therapist's guidance translates the thinking process of interactive communication into specific, intuitive, and easy-to-understand prompts in the game, which can assist participants in effectively receiving and responding to a variety of social information, reducing impulsive responses and omissions, and thereby improving the correct response rate and efficiency. 2. The therapist can interrupt or delay behaviour during the process and guide the participants' executive ability to help them make the corresponding correct actions at the right time. |
| 12 | Post-intervention outcome assessment | Assessment of symptom improvement before and after intervention | 1. Parent scale assessment: ABC, SDQ 2. Physician assessment: CARS,ADOS 3. Objective assessment: resting-state fNIRS |

**ABC, Autism Behaviour Checklist; CARS, Child Autism Rating Scale; SDQ, Strengths and Difficulties Questionnaire; ADOS, Autism Diagnostic Observation Schedule; fNIRS, functional Near-Infrared Spectroscopy.**

**Supplementary Table 2. Process of cognitive-behavioural training based on VR technology**

| Step | Content |
| --- | --- |
| 1 | The participant enters the scene and proceeds towards the designated target (red fish/red balloon/gopher), carefully observing the surroundings to identify the target, after which they step on it. |
| 2 | If the player correctly identifies the target (a red fish, red balloon, or gopher without glasses or a hat), the score will appear in green. One point will be added to the total score for each correct identification. |
| 3 | The deduction of points is indicated in red. A score of two points is deducted for stepping on a non-designated target (e.g., shark, virus, or turtle), while a score of one point is deducted for stepping on an incorrectly designated target (e.g., red fish with glasses, blue or green fish, balloon with a smiley face, or gopher with a hat or glasses). |
| 4 | When the participant earns 5 points, he/she will receive 1 Golden Dollar. The Golden Dollars will be displayed in the top left corner of the screen. As the participant plays the game following the instructions, the number of Golden Dollars increases with the score. |
